# Spatial Distribution of Dengue in a Brazilian Urban Slum Setting: Role of Socioeconomic Gradient in Disease Risk

**DOI:** 10.1371/journal.pntd.0003937

**Published:** 2015-07-21

**Authors:** Mariana Kikuti, Geraldo M. Cunha, Igor A. D. Paploski, Amelia M. Kasper, Monaise M. O. Silva, Aline S. Tavares, Jaqueline S. Cruz, Tássia L. Queiroz, Moreno S. Rodrigues, Perla M. Santana, Helena C. A. V. Lima, Juan Calcagno, Daniele Takahashi, André H. O. Gonçalves, Josélio M. G. Araújo, Kristine Gauthier, Maria A. Diuk-Wasser, Uriel Kitron, Albert I. Ko, Mitermayer G. Reis, Guilherme S. Ribeiro

**Affiliations:** 1 Centro de Pesquisas Gonçalo Moniz, Fundação Oswaldo Cruz, Salvador, Bahia, Brazil; 2 Instituto de Saúde Coletiva, Universidade Federal da Bahia, Salvador, Bahia, Brazil; 3 Escola Nacional de Saúde Pública, Fundação Oswaldo Cruz, Rio de Janeiro, Rio de Janeiro, Brazil; 4 Departamento de Microbiologia e Parasitologia, Universidade Federal do Rio Grande do Norte, Natal, Rio Grande do Norte, Brazil; 5 Department of Epidemiology of Microbial Diseases, School of Public Health, Yale University, New Haven, Connecticut, United States of America; 6 Department of Environmental Studies, Emory University, Atlanta, Georgia, United States of America; 7 Faculdade de Medicina, Universidade Federal da Bahia, Salvador, Bahia, Brazil; University of California, Davis, UNITED STATES

## Abstract

**Background:**

Few studies of dengue have shown group-level associations between demographic, socioeconomic, or geographic characteristics and the spatial distribution of dengue within small urban areas. This study aimed to examine whether specific characteristics of an urban slum community were associated with the risk of dengue disease.

**Methodology/Principal Findings:**

From 01/2009 to 12/2010, we conducted enhanced, community-based surveillance in the only public emergency unit in a slum in Salvador, Brazil to identify acute febrile illness (AFI) patients with laboratory evidence of dengue infection. Patient households were geocoded within census tracts (CTs). Demographic, socioeconomic, and geographical data were obtained from the 2010 national census. Associations between CTs characteristics and the spatial risk of both dengue and non-dengue AFI were assessed by Poisson log-normal and conditional auto-regressive models (CAR). We identified 651 (22.0%) dengue cases among 2,962 AFI patients. Estimated risk of symptomatic dengue was 21.3 and 70.2 cases per 10,000 inhabitants in 2009 and 2010, respectively. All the four dengue serotypes were identified, but DENV2 predominated (DENV1: 8.1%; DENV2: 90.7%; DENV3: 0.4%; DENV4: 0.8%). Multivariable CAR regression analysis showed increased dengue risk in CTs with poorer inhabitants (RR: 1.02 for each percent increase in the frequency of families earning ≤1 times the minimum wage; 95% CI: 1.01-1.04), and decreased risk in CTs located farther from the health unit (RR: 0.87 for each 100 meter increase; 95% CI: 0.80-0.94). The same CTs characteristics were also associated with non-dengue AFI risk.

**Conclusions/Significance:**

This study highlights the large burden of symptomatic dengue on individuals living in urban slums in Brazil. Lower neighborhood socioeconomic status was independently associated with increased risk of dengue, indicating that within slum communities with high levels of absolute poverty, factors associated with the social gradient influence dengue transmission. In addition, poor geographic access to health services may be a barrier to identifying both dengue and non-dengue AFI cases. Therefore, further spatial studies should account for this potential source of bias.

## Author Summary

Dengue is influenced by the environment; however, few studies have investigated the relationship between neighborhood characteristics and the spatial distribution of dengue within small urban areas. We examined whether specific characteristics of an urban slum community were associated with dengue risk. From January 2009 to December 2010, we conducted community-based surveillance in a slum in Salvador, Brazil to identify patients with acute febrile illness (AFI) and to test them for dengue. We identified 651 (22.0%) patients with laboratory evidence of dengue infection among 2,962 AFI patients. All the four dengue serotypes were detected, but DENV2 predominated (DENV1 8.1%; DENV2 90.7%; DENV3 0.4%; DENV4 0.8%). Estimated risk of symptomatic dengue was 21.3 and 70.2 cases per 10,000 inhabitants in 2009 and 2010, respectively. We found that neighborhood poverty level and proximity to the health center were associated with higher risk of detection of dengue and other AFI. This study highlights the large burden of dengue in poor urban slums of Brazil and indicates that socioeconomic development could potentially mitigate risk factors for both dengue and non-dengue AFI cases. In addition, we found that residential proximity to a health care facility was associated with improved case detection. Therefore, further studies on disease distribution should consider household proximity to health care facilities when assessing risk.

## Introduction

Approximately 2.5 billion people worldwide live in dengue-endemic areas and are at risk for acquiring the infection [1]. Every year, as many as 390 million dengue infections occur, resulting in an estimated 96 million symptomatic cases [2]. In the Americas, dengue incidence has continuously increased since the reintroduction of its vector, the mosquito *Aedes aegypti*, in the 1970s [3–5]. Brazil accounts for the largest number of dengue cases in the region. In 2013 alone, Brazil reported more than 1.46 million cases of dengue; 61.5% of the total number of cases recorded in the American continent [6–8].

Rapid urbanization, with subsequent increases in population density and poor living conditions, has been associated with the re-emergence of dengue [9]. Currently, approximately one third of the urban population in developing regions live in urban slums and, according to United Nations projections, about 2 billion people will reside in urban slums by 2030 [10,11]. In Brazil, a marked increase in the number of people living in impoverished urban slum communities occurred during the 20^th^ century as a consequence of intense rural to urban migration and population growth [12]. The United Nations estimated that 26.4% of Brazilians lived in slums in 2010 [13]. In Brazil and elsewhere, several studies with ecological design have found associations between increased dengue risk and demographic, socioeconomic, and environmental characteristics, such as high population and household densities [14–17], wide social inequality and low socioeconomic status [18–25], low levels of population education [24–26], presence of a precarious sanitary system [16,17], lack of garbage collection [15,18,27], and low coverage of piped water [28,29].

The majority of these studies have examined large urban areas and compared dengue occurrence among states, counties, or cities. However, dengue transmission is highly focal in space, as the vector typically disperses within a short range (<100 meters) [30,31]. Up to now, it is unknown whether group-level factors are associated with dengue at smaller geographic scales, such as within a neighborhood. In addition, prior studies, particularly those performed in Brazil, used secondary data from the national dengue reporting system. As dengue usually presents with nonspecific clinical manifestations, the disease burden may have been underreported during interepidemic periods and over-reported during epidemic periods [7], a limitation of studies using official surveillance data.

In Salvador, Brazil, dengue has been transmitted endemically since 1995, with approximately 5,000 cases reported each year between 2008 and 2012 [32,33]. We estimated the spatial distribution of symptomatic dengue in an urban slum community in Salvador, and assessed whether group-level demographic, socioeconomic, and geographic factors influenced dengue distribution. Additionally, to investigate whether any associations were specific for dengue, we repeated the spatial distribution analyses and assessed group-level associated factors using cases of non-dengue acute febrile illness (AFI) as the outcome.

## Methods

### Study site

**Fig 1:**
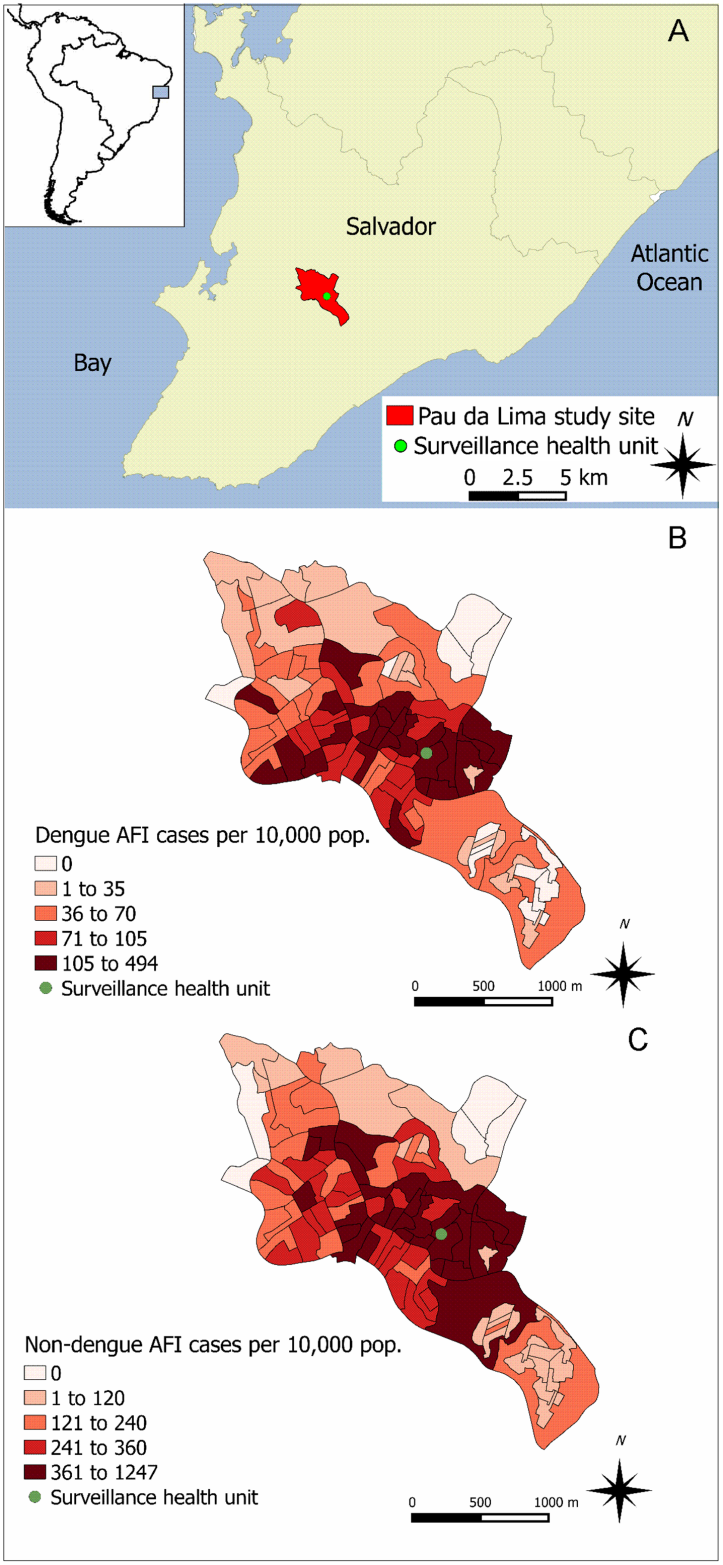
(A) Pau da Lima study site in Salvador, Brazil and spatial distribution of the estimated risk of (B) dengue and (C) non-dengue acute febrile illness (AFI) in the 98 census tracts that comprise the study site. Risks (per 10,000 population) were estimated for the two-year study period from January 1, 2009 and December 31, 2010.

Between January 1, 2009 and December 31, 2010, we conducted enhanced community-based surveillance to detect patients with laboratory evidence of dengue infection among those seeking medical care for AFI at the only public emergency health unit (São Marcos Emergency Center [SMEC]; 38°26'09"W, 12°55'32"S) serving the Pau da Lima slum community in Salvador, Brazil (Fig 1A). The study site for the community-based surveillance was arbitrarily defined to have common boundaries with census tract territories, allowing use of official social and demographic population data to determine if AFI patients who sought medical attention at SMEC lived within the study site. In 2010, we performed a community survey and found that 84% (284 of 337) of the study site residents seek medical assistance for AFI at SMEC.

According to the 2010 national census, the population of Salvador was 2.7 million and 76,352 (3%) people lived in the Pau da Lima study surveillance site [34]. The study site was comprised of 98 census tracts (CTs) in an area of 3.7 km^2^ within the Sanitary District of Pau da Lima, a delimited administrative area with a population of 218,706 in 294 CTs [34]. The site’s topography is characterized by valleys and hills, with an elevation range of 60 meters (S1 Table). Population density was >215,000 inhabitants per km^2^ for 75% of the study site’s CTs [34]. On average, 71.9% of the families living within the study area had a per capita monthly income lower or equal to the Brazilian minimum wage (R$ 510.00; equivalent to US$289.77, in 2010) (S1 Table) [34]. Demographic and socioeconomic characteristics of the study site varied among the CTs. In general, CTs located around the study health unit presented higher population densities per household and higher percentages of younger inhabitants, black population, illiteracy, and poverty (S1 Fig). Lack of sanitation was more frequent among CTs located in the northeast region of the study site (S1 Fig).

The Zoonosis Control Center at the Municipal Secretary of Health conducted vector control actions within the study site, according to the national guidelines for dengue control and prevention [35]. Vector control activities included community education on vector control measures and bimonthly household visits for entomological surveys and vector control. These actions were routinely performed throughout the study period, except for three months between August 2 and November 3, 2010, when a strike of the dengue control agents interrupted their activities. Although we informed the Pau da Lima Health District about the participants’ laboratory dengue results, we were not able to provide this information in a timely enough fashion to guide the activities of the Zoonosis Control Center agents.

### Community-based enhanced surveillance

AFI surveillance was performed at SMEC from Mondays to Fridays, from 07h30 to 16h00. During surveillance hours, the study team used medical charts to prospectively identify patients with the following inclusion criteria: age of five years or more, reported fever or measured axillary temperature ≥37.8°C of up to 21 days of duration, and household address inside the study area. Patients who agreed to participate in the study and provided informed consent had an enrollment blood sample collected and were invited to return 15 days later for convalescent-phase blood sample collection. For patients unable to return to SMEC, a study team visited their domiciles to collect convalescent-phase blood samples. Blood samples were maintained under refrigeration and were processed on the same day of collection. Sera were stored at -20°C and -70°C for dengue serological and molecular testing, respectively. The study team retrospectively reviewed medical charts of enrolled participants to collect data on presumptive diagnoses, hospitalization, and death during hospitalization at SMEC. We also reviewed medical charts for every patient attended to at SMEC in 2009 and 2010 to ascertain the number of patients who were eligible for but were not enrolled in the study. Residential addresses for enrolled patients were confirmed by household visits and their positions were marked onto hard copy 1:1,200 scale maps, which were then entered into an ArcGIS database [36]. This database was merged with a cartographical database provided by IBGE [37] to identify the CT of residence of the study patients.

### Source and definitions of study variables

CT-level aggregated data for demographic, socioeconomic, and geographic variables were obtained from the 2010 national census [34]. Topographical data were obtained from IBGE [38]. Demographic variables examined were the mean age of CT population, percentage of residents ≤15 years of age, household density in hundreds (households/100/km^2^), and population density in hundreds (inhabitants/100 km^2^). Socioeconomic variables analyzed were the percentage of households with monthly per capita income ≤1 times the national minimum wage, percentage of illiteracy among residents ≥15 years of age, mean number of residents per household, percentage of residents who self-identified as black, percentage of households with inadequate sewage disposal (households without a closed connection to the sewage system or without a closed septic tank), percentage of households without public water supply, and percentage of households not covered by a garbage collection service. We also examined the following geographic variables: mean elevation in relation to sea level, range of elevation (measured as the difference between the highest and lowest points of the CT), and two-dimensional linear distance from the CT centroid to the health unit, which was used as a proxy for health care access.

### Dengue diagnosis

Acute-phase sera were tested by enzyme linked immunoassay (ELISA) for detection of dengue NS1 antigen and dengue IgM antibodies (Panbio Diagnostics, Brisbane, Australia). Convalescent-phase sera were also tested by dengue IgM ELISA to identify seroconversions. Acute-phase sera from patients who were positive by NS1 ELISA or by IgM ELISA in either the acute- or the convalescent-phase sera were also tested by reverse transcriptase polymerase chain reaction (RT-PCR) [39] to identify the infecting serotype. We defined a dengue case as an AFI patient with a positive NS1 ELISA, acute- or convalescent-phase IgM ELISA, or RT-PCR.

### Statistical analysis

We estimated the population risk of symptomatic dengue as the ratio between the number of dengue cases detected by our surveillance and the area population. We also estimated the risk of non-dengue AFI using the number of enrolled patients without laboratory evidence of dengue as the numerator. Risks were estimated for each CT. Dengue and non-dengue AFI standardized morbidity ratios (SMR) were calculated indirectly by dividing the estimated risk for each CT during the two-year study period by the estimated risk for the overall study area. The SMRs were plotted in study site maps.

Bivariate and multivariable regression analyses to assess associations with estimated risk of dengue were performed using Poisson log-normal models [40]. This model is an extension of the Poisson model, which allows for data overdispersion. Demographic variables associated with dengue risk in the bivariate analysis (*P* value ≤0.20) were entered into a demographic multivariable backwards regression model. The same approach was used to build socioeconomic and geographic multivariable regression models. Variables with *P* values ≤0.10 in the demographic, socioeconomic, or geographic multivariable regression models were selected for entry into a final backwards Poisson log-normal model to identify significant associations (*P* ≤0.05). A conditional autoregressive model (CAR) [40,41] was then used to account for the presence of spatially correlated residuals. The CAR model is an extension of the Poisson log-normal model, with the addition of a spatial component that is dependent on the neighboring structure of the spatial units of analysis. This component assumes that neighboring areas have similar risks, which often results in a smoothed risk map. In our CAR model, we assumed adjacency-based neighborhood spatial weights. A Bayesian approach with non-informative prior distribution for all parameters was applied in the model. Calculations were made using integrated nested Laplace approximation (INLA) [42]. A backwards selection method was also applied to the CAR model to select associated variables (*P* ≤0.05). Relative risks and 95% confidence intervals (95% CI) were calculated for all the models. Model fitness was assessed by the deviance information criterion (DIC) [43]. We repeated all the steps previously described to identify demographic, socioeconomic, and geographic variables associated with the estimated risk of non-dengue AFI. Risks of dengue and non-dengue AFI, predicted by the final multivariable Poisson log-normal and by the CAR models, were used to calculate adjusted SMRs for each CT, which were plotted in maps. Statistical and spatial analyses were performed using Maptools and INLA packages in the R software (The R Project for Statistical Computing) [42]. The dataset was imported to Quantum GIS software to produce the maps [44].

### Ethics statement

This project was approved by the Research Ethics Committee at the Gonçalo Moniz Research Center, Oswaldo Cruz Foundation, the Brazilian National Council for Ethics in Research, and the Institutional Review Board of Yale University. All adult subjects provided written informed consent. Participants <18 years old who were able to read provided written assent following written consent from their parent or guardian. All study data were anonymized before analysis.

## Results

**Fig 2:**
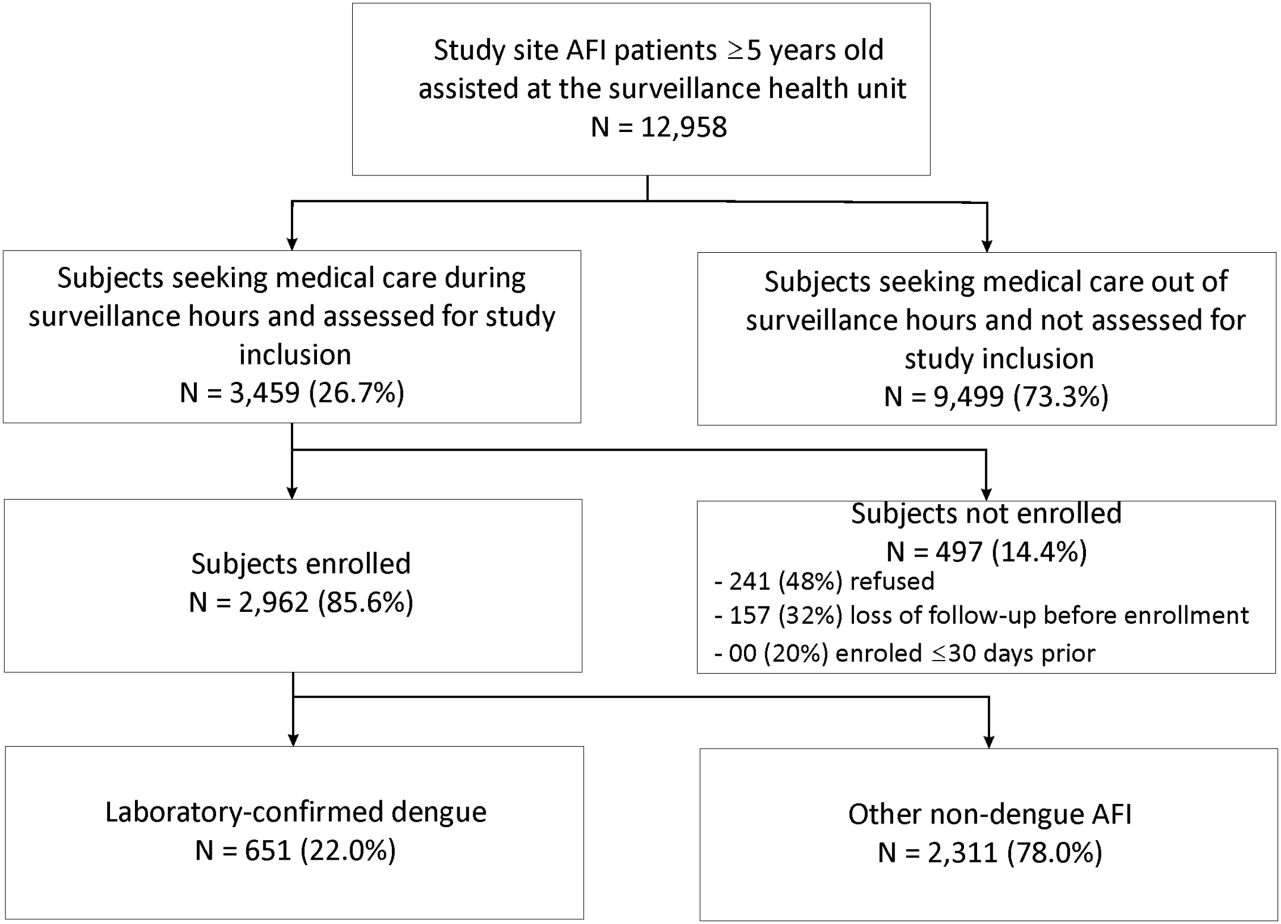
Enrollment of acute febrile illness (AFI) patients and dengue detection through enhanced surveillance in the Pau da Lima community, Salvador, Brazil, from January 1, 2009 to December 31, 2010.

During the two-year study period, a total of 12,958 study site residents ≥5 years old received medical care for an AFI at SMEC. Among these residents, 3,459 (26.7%) were evaluated for study inclusion (Fig 2). Age and sex distributions for the groups of patients who were and were not evaluated were similar (both groups had median age of 18 years old and were 47% male). Of the assessed patients, 2,962 (85.6%) were enrolled in the study. Patients who were enrolled in the study were older (19 versus 13 years) and were more likely to be male (48% versus 44%) compared to those not enrolled.

An acute-phase blood sample was collected from 2,874 (97.0%) enrolled patients. Paired blood samples were obtained from 2,523 (85.2%) patients. Laboratory testing identified 651 (22.0% of 2,962) patients with evidence of dengue infection; the remaining 2,311 (78.0%) patients were classified as having non-dengue AFI. Among the dengue cases, 380 (58.4%) were acute-phase IgM ELISA positive, 505 (77.6%) were convalescent-phase IgM ELISA positive, 103 (15.8%) were NS1 ELISA positive, and 247 (37.9%) were RT-PCR positive. IgM seroconversion was observed for 207 (31.8%) of the dengue cases. For RT-PCR confirmed cases, 20 (8.1%) were infected with DENV1, 224 (90.7%) with DENV2, 1 (0.4%) with DENV3, and 2 (0.8%) with DENV4. Dengue was less prevalent among patients enrolled in 2009 (152 of 1,466; 10.4%) compared to patients enrolled in 2010 (499 of 1,496; 33.4%). The estimated risk of symptomatic dengue in the study site was 21.29 and 70.23 cases per 10,000 inhabitants in 2009 and 2010, respectively.

The socio-demographic and clinical characteristics of dengue and non-dengue AFI patients are shown in Table 1. Compared to non-dengue AFI, dengue cases more frequently presented with myalgia, retro-orbital pain, arthralgia, and rash. Only 16% of the dengue cases had a presumptive diagnosis of dengue recorded in their medical charts. Yet, the likelihood of dengue suspicion was 7.5 times higher among dengue cases than among non-dengue AFI patients (P <0.001).

**Table 1:** Socio-demographic and clinical characteristics of acute febrile illness (AFI) patients detected through enhanced surveillance in the Pau da Lima community, Salvador, Brazil, according to dengue laboratory testing results—January 1, 2009 to December 31, 2010.

Characteristics	Dengue	Non-dengue AFI	Total
	Number/Total (%) or Median (Interquartile Range)		
**Socio-demographic**			
Male gender^a^	339/651 (52%)	1,069/2,311 (46%)	1,408/2,962 (48%)
Age, in years^b^	16 (10–29)	19 (10–30)	19 (10–30)
Self-reported race			
Black	278/569 (49%)	1,071/2,133 (50%)	1,349/2,702 (50%)
Mixed	224/569 (39%)	827/2,133 (39%)	1,051/2,702 (39%)
White	50/569 (9%)	196/2,133 (9%)	246/2,702 (9%)
Other	17/569 (3%)	39/2,133 (2%)	56/2,702 (2%)
Illiterate participants aged ≥ 15 years	5/352 (1%)	27/1,374 (2%)	32/1,726 (2%)
**Clinical**			
Days of symptoms^c^	3 (2–4)	3 (2–4)	3 (2–4)
Headache	553/645 (86%)	1,912/2,299 (83%)	2,465/2,944 (84%)
Prostration	500/639 (78%)	1,818/2,264 (80%)	2,318/2,903 (80%)
Myalgia^a^	472/644 (73%)	1,588/2,298 (69%)	2,060/2,942 (70%)
Retro-orbital pain^a^	313/643 (49%)	832/2,290 (36%)	1,145/2,933 (39%)
Arthralgia^a^	279/639 (44%)	756/2,287 (33%)	1,035/2,926 (35%)
Rash^a^	148/645 (23%)	313/2,299 (14%)	461/2,944 (16%)
Vomiting	197/645 (31%)	676/2,303 (29%)	873/2,948 (30%)
**Outcome** ^d^			
Clinical suspicion of dengue^a^	102/648 (16%)	46/2,288 (2%)	148/2,936 (5%)
Hospitalization	20/648 (3%)	48/2,288 (2%)	68/2,936 (2%)
Death	1/648 (<1%)	3/2,288 (<1%)	4/2,936 (<1%)

^a^Two-tailed chi-square P value <0.05, dengue versus non-dengue AFI patients.

^b^Data on age were available for 2,961 AFI enrolled patients (651 with dengue and 2,310 with other non-dengue AFI).

^c^Data on days of symptoms prior to presentation were available for 2,943 AFI enrolled patients (643 with dengue and 2,300 with other non-dengue AFI).

^d^Outcome determined by medical chart review.

We were able to locate the census tract of residence for 570 (87.6%) of the 651 dengue cases and for 1,948 (84.3%) of the 2,311 non-dengue AFI patients. The estimated risks for both dengue and non-dengue AFI were higher for the population living in the census tracts located in the central region of the study site (Fig 1B and 1C).

Multivariable Poisson log-normal models identified the following CT-level factors associated with increased risk of dengue: a shorter linear distance between the centroid of the census tract and the emergency unit, a higher percentage of inhabitants who self-identify as black, and a higher percentage of families earning lower or equal to one Brazilian minimum wage per household inhabitant per month (Table 2). Estimated risk for non-dengue AFI was independently associated with the same CT-level factors, with higher mean age of the CT population as an additional risk factor (Table 2).

**Table 2:** Factors associated with dengue and non-dengue acute febrile illness (AFI) measured by Poisson log-normal models (bivariate and multivariable) and conditional auto-regressive model (spatial), Pau da Lima community, Salvador, Brazil—January 1, 2009 to December 31, 2010.

Characteristics	Dengue			Non-dengue AFI		
	Bivariate	Multivariable^a^	Spatial^b^	Bivariate	Multivariable^c^	Spatial^d^
	Relative Risk (95%CI)			Relative Risk (95%CI)		
**Demographics**						
Population density (x100 inhabitants/km^2^)	1.00 (0.99–1.00)			1.00 (0.99–1.00)		
Household density (x100 households/km^2^)	1.00 (0.99–1.00)			1.00 (0.99–1.00)		
Percentage of inhabitants <15 years of age	**1.11 (1.07–1.16)**			**1.07 (1.04–1.11)**		
Mean age	**0.84 (0.78–0.90)**			**0.90 (0.84–0.96)**	**1.16 (1.08–1.25)**	**1.10 (1.02–1.19)**
**Socioeconomic**						
Percentage of black population	**1.07 (1.05–1.09)**	**1.02 (1.01–1.04)**		**1.06 (1.04–1.08)**	**1.03 (1.02–1.05)**	**1.02 (1.01–1.04)**
Percentage of illiterates	**1.13 (1.08–1.18)**			**1.10 (1.05–1.15)**		
Percentage density per household	**5.29 (2.79–10.31)**			**4.15 (2.32–7.45)**		
Percentage of households:						
With per capita monthly income ≤1 minimum wage^e^	**1.04 (1.03–1.05)**	**1.02 (1.01–1.03)**	**1.02 (1.01–1.04)**	**1.03 (1.02–1.03)**	**1.03 (1.02–1.04)**	**1.03 (1.01–1.04)**
With inadequate sewer disposal	1.00 (0.99–1.01)			1.00 (0.99–1.01)		
Without public water supply	0.98 (0.92–1.04)			0.97 (0.92–1.03)		
Without garbage collection	1.02 (1.00–1.04)			1.01 (0.99–1.04)		
**Geographic**						
Mean elevation (m)	**0.98 (0.96–0.99)**			**0.98 (0.97–0.99)**		
Elevation range (m)	**1.03 (1.01–1.04)**			1.01 (1.00–1.03)		
Distance from CT centroid to SMEC (x100 m)	**0.87 (0.84–0.91)**	**0.92 (0.89–0.95)**	**0.87 (0.80–0.94)**	**0.87 (0.84–0.90)**	**0.91 (0.88–0.93)**	**0.87 (0.80–0.93)**

^a^ Deviance information criteria (DIC) = 456.1

^b^ DIC = 443.1

^c^ DIC = 632.2

^d^ DIC = 609.8

^e^R$ 510.00; equivalent to US$289.77, in 2010

**Fig 3:**
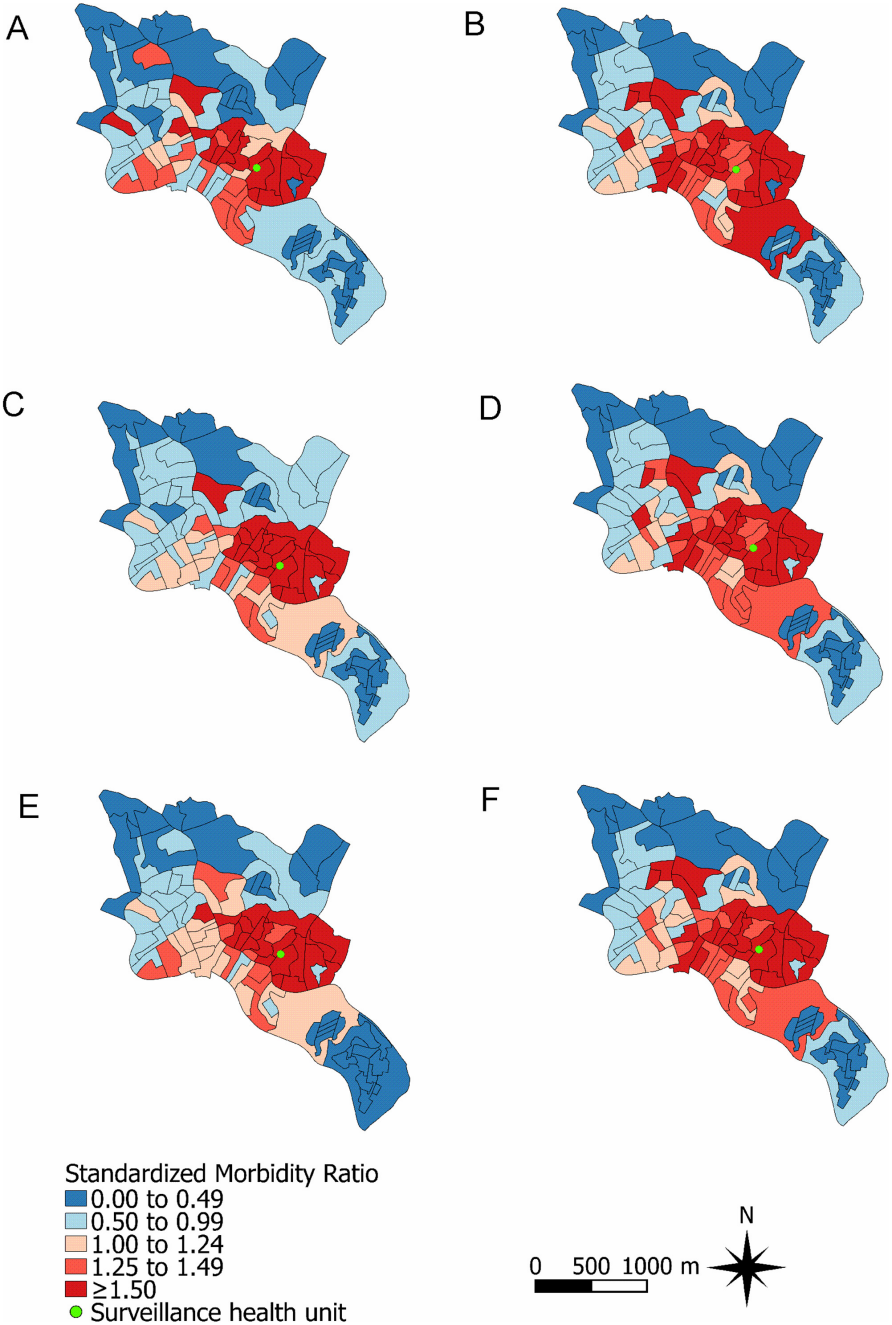
Standardized morbidity ratios (SMRs) for dengue and non-dengue acute febrile illness (AFI) in the Pau da Lima community, Salvador, Brazil, from January 1, 2009 to December 31, 2010. Non-adjusted SMR for (A) dengue and (B) non-dengue AFI; SMR adjusted by the final Poisson-log normal model for (C) dengue and (D) non-dengue AFI; SMR adjusted by the final conditional auto-regressive model for (E) dengue and (F) non-dengue AFI.

Addition of a spatial component to the multivariable models for both dengue and non-dengue AFI improved their fitness and seemed to capture the spatial pattern of dengue and non-dengue AFI, since the non-structured residuals of both models were randomly distributed in space. Dengue risk, assessed by the CAR model, increased 2% (RR: 1.02; 95% CI: 1.01–1.04) for each 1% increase in the percentage of CT families with a monthly income ≤1 times the Brazilian minimum wage per household inhabitant, and decreased 13% (RR: 0.87; 95% CI: 0.80–0.94) for each 100 meter increase in the linear distance between the CT centroid and the surveillance health unit (Table 2). Non-dengue AFI risk also increased as the percentage of families with a monthly income of ≤1 times the minimum wage per household inhabitant increased (RR: 1.03; 95% CI: 1.01–1.04) and decreased as the linear distance between the CT centroid and the surveillance health unit increased (RR: 0.87; 95% CI: 0.80–0.93). In addition, non-dengue AFI was positively associated in the CAR model with higher mean age of the CT population (RR: 1.10; 95% CI: 1.02–1.19), and with a higher percentage of inhabitants who are black (RR: 1.02; 95% CI: 1.01–1.04) (Table 2). Although the spatial distribution of SMRs adjusted by the final Poisson log-normal and CAR models were smoother than the non-adjusted SMRs for both dengue and non-dengue AFI, the CTs located in the central region of the study site maintained a higher relative risk for both conditions (Fig 3).

## Discussion

This enhanced surveillance study highlights the large burden of symptomatic dengue in a poor urban slum community of Salvador, the third largest city in Brazil. Even though the study site was relatively small and characterized by high levels of absolute poverty, the spatial distribution of the detected dengue cases was not homogenous, being influenced by neighborhood characteristics; namely, the gradient of social status and proximity to health services. These findings were not specific for dengue, as the spatial distribution of non-dengue AFI presented the same pattern.

During the study period, the case definition for suspected dengue in Brazil was a patient who lived or traveled to endemic areas and presented with fever up to seven days of duration plus two of the following symptoms: headache, retro-orbital pain, myalgia, arthralgia, rash, or prostration [45]. However, underreporting of patients fulfilling clinical and epidemiological criteria for dengue is common in Brazil and elsewhere [46,47]. Furthermore, dengue reporting tends to be influenced by disease severity and the availability of dengue laboratory testing [48]. We used an enhanced surveillance design to detect AFI patients with laboratory evidence of dengue infection. This approach allowed identification of dengue cases that were unlikely to be reported, as only 16% of the detected cases had a clinical suspicion of dengue recorded in their chart, and also provided more complete epidemiological disease data.

Enhanced surveillance was only conducted during working hours, resulting in an underestimation of dengue and other non-dengue AFI risks. Furthermore, enrolled patients were older than those evaluated but not enrolled, which also might have influenced dengue risk estimation as dengue risk is not equal for all age groups. However, compared to the incidence of reported dengue in the whole Sanitary District of Pau da Lima, the study detected greater dengue risk in 2009 (17.3 and 21.3 cases per 10,000 inhabitants, respectively) and in 2010 (44.1 and 70.2 cases per 10,000 inhabitants, respectively) [33]. This finding is noteworthy, since the study only enrolled 22.9% of the 12,958 AFI subjects from the study site seeking medical attention at SMEC. As the AFI patients who were assessed for study inclusion had comparable age and sex distribution to those not assessed, we can assume that dengue prevalence was similar between these two groups and infer that the actual risk for a dengue episode requiring medical attention in the study site was about four times greater than estimated.

We found a higher risk of dengue associated with poorer areas in the Pau da Lima slum community. Although some population-level studies based on reported dengue cases have also shown an association of symptomatic dengue risk and lower socioeconomic status [21–23,49], others have found an inverse association, where greater incidence occurred in areas of higher income [25,50], or even no association [51]. Discrepancies have been observed in individual-level studies, where dengue occurrence has not been associated [52,53] or was positively [54–56] or negatively [24,57] associated with income and socioeconomic status. It has been speculated that these contradictory results were due to the specificities of each study location, such as level of dengue susceptibility in the population, implementation and coverage of vector control measures, as well as differences in the study spatial unit or the socioeconomic variables considered [18,22,29]. However, poor communities typically have environmental characteristics that facilitate *Aedes spp*. breeding, including presence of refuse deposits and containers for water storage [58,59]. Therefore, the social gradient we found in association with increased risk of dengue may have acted as a surrogate for other proximal factors involved in dengue transmission.

Proximity of the CTs to the health unit was the variable most strongly associated with detection of dengue. This finding may be due to the fact that CTs located around the health unit had higher population densities per household, and higher percentage of inhabitants <15 years of age (a proxy for susceptibility to dengue infection) (S1 Fig); together these facts might favor dengue transmission as they increase opportunities for interactions between infected and susceptible hosts via the mosquito vector. In bivariate, but not in multivariable and CAR analyses, both population density per household, and percentage of inhabitants <15 years of age were associated with dengue detection. However, the distance between the CTs and the health unit was also positively associated with non-dengue AFI cases detection, suggesting that this association was not specific for a vector-born disease. Therefore, CTs proximity to the study health unit most likely indicates increased opportunity for case detection. Measured distances between households and health facilities have previously been associated with dengue occurrence [28], as well as with poorer colorectal cancer survival [60], lower clinic attendance and a higher degree of dehydration due to diarrhea [61], and decreased use of antenatal healthcare [62], among others. Geographic accessibility to health care is usually observed on a broader scale, especially in developing countries where greater inequalities in health care access are observed in smaller towns distant to large urban centers [63,64]. Our study demonstrates that this phenomenon may also be present at finer geographic scales, such as within urban communities. This finding may be particularly important in spatial distribution studies that use reported cases of mild and self-limited diseases, and that rely on passive surveillance. In this context, areas of higher disease risk may actually represent areas of greater provision of health services and greater opportunity for case detection rather than a true difference in disease frequency.

Other studies have identified a higher occurrence of dengue in areas that lack or have infrequent garbage collection [15,27,65], that have a lower coverage of closed sewer systems [17], and those with low coverage or irregular water supplies [28,57,66]. These associations may be explained by ecological preferences of the mosquito vectors, which find more favorable larval development sites in areas with poorer sanitation infrastructure. In bivariate analysis, we found an association between increased dengue risk and inadequate garbage collection; however, a significant independent association was not observed after adjusting for other covariates. We were unable to identify associations with the coverage levels of piped water supply and sewer provision. The divergence between our findings and those from other studies may be explained by the low variability in the characteristics of the CTs comprising our study site, or by colinearity with other socioeconomic variables included in the model (S2 Table). Alternatively, the inclusion of the distance from the health care unit and the CT centroid in the model may have overshadowed weaker associations.

This study has several limitations. Despite SMEC being the sole public emergency unit in the community, with the second closest public emergency unit located >1.5 km outside from the study site’s boundaries, Pau da Lima residents may have sought care elsewhere. In addition, we were not able to investigate dengue in all AFI patients seeking medical assistance at SMEC. However, AFI patients who were and were not evaluated for study inclusion were similar regarding age and sex distribution, suggesting that selection bias had a minor influence on our results. The CT of residence was not identified for all study participants, but we georeferenced the majority of them (87.6%) and ensured accuracy of the locations of CTs through household visits. We used different laboratory approaches to identify dengue cases. Even though it is likely that we missed some dengue cases by only performing RT-PCR on patients who were NS1 or IgM ELISA positive, the method we used to simultaneous test dengue by IgM and NS1 assays has been shown to increase diagnostic sensitivity [67]. Use of IgM ELISA to confirm dengue is consistent with the Brazilian Ministry of Health guidelines [68,69]; however, dengue IgM may remain detectable up to two months after an infection, and we may have classified patients with recent dengue infection as dengue cases. To account for the possible inclusion of recent asymptomatic infections among dengue cases, we repeated the multivariable Poisson-log normal and the CAR regression analyses using only patients confirmed by IgM ELISA seroconversion, NS1 ELISA, and RT-PCR and found similar associations (S3 Table). Finally, in our model, we could not include data from the Larval Index Rapid Assay for *Aedes aegypti* (LIRAa), a national survey for positive mosquito breeding sites in a random sample of dwellings [70], because this index is recorded in spatial units that do not align with CTs boundaries.

Strengths of this study include the laboratory testing of all enrolled patients and the assessment of group-level characteristics associated with non-dengue AFI. Additionally, we used conditional auto-regressive models, which increased model fitness by adding a spatially structured component. The increases in model fit indicate that there were residual spatial variations in the risk distributions that had not initially been captured by the studied variables.

Official surveillance systems based on passive reporting underestimate dengue burden; thus, enhanced surveillance is a useful tool to provide more accurate estimates of disease occurrence and its spatial distribution. According to the World Health Organization guidelines for dengue prevention and control, estimating the true burden of the disease is a critical step to achieve the goal of reducing dengue disease burden [71]. Our findings corroborate those of other studies showing that implementation of sentinel health unit-based enhanced surveillance for dengue is feasible and may be employed to obtain high quality information on disease trends and circulating serotypes as well as increase opportunities for timely detection and intervention during epidemics, which may not be achieved by passive surveillance [46,72].

In several settings, low socioeconomic status has been observed to impact dengue transmission [21,54,73], emphasizing that the disease burden is likely to be greatest in vulnerable populations such as urban slum dwellers, and as we found in this study, the poorest segments of such populations. Until initiatives address social inequity and the underlying poverty-associated environmental determinants of dengue transmission, specific vector control actions that are difficult to apply citywide, such as biological larvae control, may target groups at higher disease risk, such as those living in the poorer areas of urban communities.

Finally, studies aiming to assess spatial distribution and group-level associated factors of diseases should account for potential detection bias. With the popularization of GIS and spatial analysis tools, the distance between each area unit and the closest health service is a viable proxy for health care accessibility and its use may help explain the spatial distribution of health and disease.

## Supporting Information

S1 Table Characteristics of the 98 census tracts (CTs) comprising the study area in Pau da Lima, Salvador, Brazil, according to the 2010 national census. (DOCX)

S2 Table Spearman's rank correlation coefficient between socioeconomic variables. (DOCX)

S3 Table Factors associated with laboratory confirmed dengue measured by Poisson log-normal models (bivariate and multivariable) and conditional auto-regressive model (spatial), Pau da Lima community, Salvador, Brazil—January 1, 2009 to December 31, 2010. (DOCX)

S1 Fig Spatial distribution of demographic, socioeconomic and geographical characteristics of Pau da Lima study site in Salvador, Brazil, according to the 2010 national census. (A) Population density in hundreds (inhabitants/100/km2), (B) Population density per household, (C) percentage of inhabitants <15 years of age, (D) percentage of black population, (E) percentage of illiterates, (F) percentage of households with per capita monthly income ≤1 minimum wage (R$ 510.00; equivalent to US$289.77, in 2010), (G) percentage of households with inadequate sewer disposal, (H) percentage of households without public water supply, (I) percentage of households without garbage collection and (J) distance from CT centroid to SMEC (x100 m).
(TIF)

S1 Database Database containing the numbers of confirmed and probable dengue cases and the numbers of non-dengue AFI cases, per study year and census tract. (XLS)

S1 Checklist STROBE checklist. (DOC)
