# Supplementary material for: Spatial Distribution of Dengue in a Brazilian Urban Slum Setting: Role of Socioeconomic Gradient in Disease Risk
Source: PLoS Negl Trop Dis. 2015 Jul 21;9(7):e0003937. doi: 10.1371/journal.pntd.0003937 (PMC4510880; doi:10.1371/journal.pntd.0003937)
Supplement: S1 Table — (DOCX) [file pntd.0003937.s001.docx]

**S1 Table. Characteristics of the 98 census tracts (CT) comprising the Pau da Lima study site, in Salvador, Brazil, according to the 2010 national census.**

| **Characteristics** | | **Mean** | **Standard Deviation** | **Median** | **Interquartile range** | **Minimum-Maximum** |
| --- | --- | --- | --- | --- | --- | --- |
| **Demographics** | |  |  |  |  |  |
| Residents | | 779.1 | 264.8 | 756.5 | 572.8 - 924.0 | 290 - 1.723 |
| Number of households | | 251.6 | 89.1 | 234.0 | 190.8 - 295.8 | 93.0 - 619.0 |
| Population density (x100 inhabitants/km^2^) | | 330.8 | 180.5 | 324.0 | 217.4 - 407.2 | 19.3 - 1.204.0 |
| Household density (x100 households/km^2^) | | 108.8 | 65.1 | 101.8 | 63.4 - 133.8 | 5.8 - 368.8 |
| Proportion of inhabitants <15 years of age | | 22.8 | 5.3 | 22.8 | 21.0 - 26.3 | 11.0 - 34.7 |
| Mean age | | 30.0 | 2.7 | 29.9 | 28.2 - 31.7 | 24.1 - 36.2 |
| **Socioeconomic** | |  |  |  |  |  |
| Proportion of black population | | 31.6 | 8.8 | 31.8 | 24.2 - 37.9 | 15.1 - 57.4 |
| Proportion of illiterate (among those ≥15 years of age) | | 5.5 | 3.8 | 4.9 | 2.8 - 7.1 | 0.0 - 16.1 |
| Proportion of households: | |  |  |  |  |  |
|  | With per capita monthly income ≤ 1 minimum wage^a^ | 71.9 | 20.7 | 78.3 | 71.5 - 86.0 | 21.6 - 95.8 |
|  | With inadequate sewer disposal | 15.8 | 26.4 | 2.0 | 0.4 - 18.4 | 0.0 - 98.2 |
|  | Without public water supply | 1.2 | 3.3 | 0.3 | 0.0 - 1.1 | 0.0 - 27.8 |
|  | Without garbage collection | 4.2 | 8.5 | 0.4 | 0.0 - 4.5 | 0.0 - 47.7 |
| Population density per household | | 3.1 | 0.3 | 3.2 | 2.9 - 3.3 | 2.5 - 3.9 |
| **Geographic** | |  |  |  |  |  |
| Area (1000 m^2^) | | 38.0 | 45.9 | 24.9 | 15.7 – 38.3 | 6.9 – 277.70 |
| Mean elevation (m) | | 64.1 | 11.1 | 65.0 | 55.6 - 71.6 | 35.9 - 93.5 |
| Elevation range (m) | | 33.4 | 13.3 | 35.0 | 29.7 - 41.9 | 1.43 - 60.0 |
| Distance from CT centroids to SMEC (x100 m) | | 8.2 | 4.3 | 7.8 | 4.5 - 11.4 | 0.7 - 19.5 |

SMEC = São Marcos Emergency Center

^a^ R$ 510.00; equivalent to US$289.77, in 2010.
