# Supplementary material for: Spatial Distribution of Dengue in a Brazilian Urban Slum Setting: Role of Socioeconomic Gradient in Disease Risk
Source: PLoS Negl Trop Dis. 2015 Jul 21;9(7):e0003937. doi: 10.1371/journal.pntd.0003937 (PMC4510880; doi:10.1371/journal.pntd.0003937)
Supplement: S2 Table — (DOCX) [file pntd.0003937.s002.docx]

**S2Table. Spearman’s rank correlation coefficient between socioeconomic variables.**

| **Variables** | Percentage of black population | Percentage of illiterates | Population density per household | Percentage of households with per capita monthly income ≤1 minimum wage^a^ | Percentage of households with inadequate sewer disposal | Percentage of households without public water supply | Percentage of households without garbage collection |
| --- | --- | --- | --- | --- | --- | --- | --- |
| Percentage of black population | 1.00 | - | - | - | - | - | - |
| Percentage of illiterates | 0.68 | 1.00 | - | - | - | - | - |
| Population density per household | 0.58 | 0.64 | 1.00 | - | - | - | - |
| Percentage of households with per capita monthly income ≤1 minimum wage^a^ | 0.58 | 0.75 | 0.73 | 1.00 | - | - | - |
| Percentage of households with inadequate sewer disposal | 0.42 | 0.64 | 0.52 | 0.56 | 1.00 | - | - |
| Percentage of households without public water supply | 0.12 | 0.36 | 0.21 | 0.22 | 0.40 | 1.00 | - |
| Percentage of households without garbage collection | 0.07 | 0.22 | 0.19 | 0.14 | 0.34 | 0.27 | 1.00 |

^a^R$ 510.00; equivalent to US$289.77, in 2010
