# Supplementary material for: Spatial Distribution of Dengue in a Brazilian Urban Slum Setting: Role of Socioeconomic Gradient in Disease Risk
Source: PLoS Negl Trop Dis. 2015 Jul 21;9(7):e0003937. doi: 10.1371/journal.pntd.0003937 (PMC4510880; doi:10.1371/journal.pntd.0003937)
Supplement: S3 Table — (DOCX) [file pntd.0003937.s003.docx]

**Table S3. Factors associated with confirmed dengue measured by Poisson log-normal models (bivariate and multivariable) and conditional auto-regressive model (spatial), Pau da Lima community, Salvador, Brazil - January 1, 2009 to December 31, 2010.**

| **Characteristics** | | **Dengue** | | |
| --- | --- | --- | --- | --- |
|  |  | **Bivariate** | **Multivariable^a^** | **Spatial^b^** |
|  |  | **Relative Risk (95%CI)** | | |
| **Demographics** | |  |  |  |
| Population density (x100 inhabitants/km^2^) | | 1.00 (0.99-1.00) |  |  |
| Household density (x100 households/km^2^) | | 1.00 (0.99-1.00) |  |  |
| Percentage of inhabitants <15 years of age | | **1.10 (1.06-1.15)** |  |  |
| Mean age | | **0.85 (0.78-0.91)** |  |  |
| **Socioeconomic** | |  |  |  |
| Percentage of black population | | **1.06 (1.04-1.08)** |  |  |
| Percentage of illiterates | | **1.12 (1.07-1.18)** |  |  |
| Percentage density per household | | **4.55 (2.31-9.22)** |  |  |
| Percentage of households: | |  |  |  |
|  | With per capita monthly income ≤1 minimum wage^c^ | **1.03 (1.02-1.05)** | **1.02 (1.01-1.04)** | **1.02 (1.01-1.04)** |
|  | With inadequate sewer disposal | 1.00 (1.00-1.01) |  |  |
|  | Without public water supply | 0.95 (0.88-1.03) |  |  |
|  | Without garbage collection | 1.02 (1.00-1.04) |  |  |
| **Geographic** | |  |  |  |
| Mean elevation (m) | | **0.98 (0.96–0.99)** |  |  |
| Elevation range (m) | | **1.02 (1.01-1.04)** |  |  |
| Distance from CT centroid to SMEC (x100 m) | | **0.87 (0.83-0.90)** | **0.90 (0.86-0.93)** | **0.85 (0.78-0.92)** |

^a^ Deviance information criteria (DIC)= 380.3

^b^ DIC= 369.4

^c^R$ 510.00; equivalent to US$289.77, in 2010
